# Development of a small batch flexible station for packaging waste sorting in circular economy applications

**DOI:** 10.3389/frobt.2026.1870171

**Published:** 2026-10-09

**Authors:** Branko Lukić, Miloš Petrović, Nikola Knežević, Zaviša Gordić, Arso M. Vukićević, Kosta Jovanović

**Affiliations:** 1 School of Electrical Engineering, University of Belgrade, Belgrade, Serbia; 2 Faculty of Engineering, University of Kragujevac, Kragujevac, Serbia

**Keywords:** circular economy, collaborative robotics, computer vision, deep learning, small-batch production, waste sorting

## Abstract

**Introduction:**

Efficient packaging waste sorting is an important requirement for implementing circular economy strategies, yet many automated sorting solutions are designed for large-scale, high-throughput recycling facilities and are not well suited to small-batch industrial environments characterized by limited waste volumes, frequent material changes, and the need for rapid system reconfiguration.

**Methods:**

This paper presents the design, implementation, and laboratory validation of a modular collaborative robotic workstation for small-batch packaging waste sorting. The proposed system integrates a collaborative robot, RGB-D vision system, conveyor-based material flow, and a ROS2-based control architecture within a unified perception–decision–manipulation framework. The perception pipeline combines class-agnostic segmentation using the Segment Anything Model (SAM) with lightweight MobileNetV2-based classification for material recognition and is extended with optional modules for geometric reasoning and damage assessment. The proposed architecture reduces dependence on task-specific segmentation training and provides semantic information relevant to grasp planning, safety-aware task allocation, and human–robot collaboration.

**Results:**

The workstation was validated under controlled laboratory conditions using three representative packaging waste categories: plastic bottles, aluminium cans, and Tetra Pak containers. The SAM + MobileNetV2 perception pipeline achieved an accuracy of 0.93, precision of 0.93, recall of 0.92, and F1-score of 0.92. System-level experiments demonstrated successful integration of RGB-D object localization, conveyor tracking, camera-to-robot coordinate transformation, and robotic pick-and-place execution. In contrast to conventional high-throughput sorting systems, the proposed workstation emphasizes modularity, flexibility, and collaborative operation for small-batch industrial packaging waste streams. In addition, workstation-level manipulation tests over 200 grasp attempts resulted in a 92.0% grasp success rate, an 89.0% end-to-end sorting success rate, and an estimated sorting rate of 5.73 picks/min under controlled conveyor-based conditions.

**Discussion:**

The results demonstrate the feasibility of integrating foundation-model-based perception with collaborative robot in a modular workstation intended for small-batch industrial packaging waste sorting. The proposed approach emphasizes flexibility, modularity, and reduced reconfiguration effort for variable waste streams. The results provide a basis for further development toward adaptive and human–robot collaborative sorting, while broader validation under more diverse, cluttered, and industrially representative conditions remains necessary.

## 1 Introduction

Waste generation is an undesired outcome of modern production systems and intensified consumption habits, making efficient waste management a fundamental prerequisite for sustainable development and environmental protection. Global municipal solid waste production is projected to reach approximately 3.40 billion tons by 2050 increasing from 2.01 billion tons in 2018 (Kaza et al., 2018), posing a significant environmental burden while simultaneously representing a valuable resource within circular economy paradigms. The concept of the circular economy emphasizes extending material lifecycles and minimizing waste generation by enabling reuse and remanufacturing processes. Therefore, in this context, landfilling is considered the least desirable option, whereas reuse, recycling, and recovery are prioritized to retain material value within the production cycle. A key enabler of circular economy systems is timely and accurate waste sorting, which directly impacts recycling efficiency and reduces the volume of waste directed to landfills. Low effective waste management threatens the achievement of several United Nations Sustainable Development Goals (SDGs) (United Nations, 2015), while improved waste handling can lower production costs (Korhonen et al., 2018), and decrease energy dependency (Olives et al., 2023). These benefits are particularly relevant for industrial enterprises, where waste management directly influences production costs and resource utilization.

Despite the widespread adoption of modern manufacturing philosophies such as Lean, which promote “zero waste” as a key objective (Afum et al., 2022), complete elimination of waste remains unrealistic in practice (Quicker et al., 2020). Instead, industries focus on minimizing waste generation and maximizing reuse. Nevertheless, a fraction of materials inevitably remains and must be sorted for recycling or disposal. Although the waste sorting industry has seen significant levels of automation, a portion of waste, typically at the end of sorting lines, still requires manual labour. These tasks involve a limited number of object categories, distinguishing industrial waste sorting from municipal waste sorting, which often requires classification of hundreds of materials. With the increasing adoption of small-batch and customized manufacturing, there is a growing need for flexible production systems capable of adapting to changing conditions with minimal reconfiguration. In this work, packaging waste sorting is considered in the context of controlled industrial and small-batch production environments rather than fully mixed municipal waste management, where waste streams are typically more heterogeneous, contaminated, densely cluttered, and difficult to precondition. Therefore, the proposed workstation targets industrial packaging waste streams in which flexibility, rapid reconfiguration, and safe human–robot collaboration are prioritized over maximum throughput.

Robotic waste sorting systems, typically based on pick-and-place architectures, integrate conveyor systems, vision modules, and robotic manipulators to identify and separate objects from waste streams (Koskinopoulou et al., 2021; Kiyokawa et al., 2021). A typical system consists of a conveyor, a camera-based perception system, and a robot executing sorting operations. A key challenge lies in the ability of vision algorithms to recognize diverse waste items and in the robustness of grippers handling irregular shapes (Zeng et al., 2017). Numerous studies have explored such systems using deep learning and computer vision, demonstrating high classification accuracy in controlled environments but also highlighting limitations in generalization to new object categories (Lin et al., 2021; Kiyokawa et al., 2021). Additionally, multi-sensor fusion approaches incorporating RGB, depth, and near-infrared data have been proposed to improve material recognition, albeit at the cost of increased system complexity (Xiao et al., 2020).

Recent advances in artificial intelligence, particularly deep learning, have significantly improved object detection and classification. However, most approaches rely on supervised learning techniques such as CNNs and YOLO (Redmon et al., 2016; Krizhevsky et al., 2012), requiring large annotated datasets and retraining for new object types. This dependency has been widely reported as a key limitation in robotic waste sorting systems, particularly in dynamic industrial environments (Gundupalli et al., 2017).

Recent developments in foundation models such as the Segment Anything Model (SAM) (Kirillov et al., 2023) offer a promising alternative by enabling class-agnostic segmentation without extensive training data. This is particularly important in small-batch environments, where frequent changes in waste composition make dataset-driven retraining impractical.

Despite significant progress in automated waste sorting systems, most existing solutions are designed for high-throughput, large-scale facilities, where economic justification relies on continuous operation and large waste volumes (Boschetti et al., 2025), making them unsuitable for small-batch industrial environments characterized by high variability, frequent product changes, and limited waste quantities. This creates a technological gap in developing flexible sorting solutions tailored to small-scale applications, for which economic feasibility must be assessed under application-specific conditions. Existing industrial systems, such as those developed by AMP Robotics and ZenRobotics, primarily focus on high-capacity automated sorting using large-scale installations (AMP Robotics, 2026; Lukka et al., 2014), and while highly efficient, they lack the flexibility required for small-batch scenarios and involve significant capital investment. In contrast, the approach proposed in this paper emphasizes modularity and adaptability rather than throughput maximization, highlighting the need for a flexible waste sorting station capable of handling small batches of heterogeneous waste streams while maintaining adaptability and the potential for simplified reconfiguration. The proposed small-batch solution aligns with Industry 4.0 and Industry 5.0 principles, as it emphasizes flexibility, reconfigurability, and intelligent automation. Furthermore, challenges such as robust robotic grasping of irregular and deformable waste items remain critical, as highlighted in recent studies on data-driven grasping and manipulation strategies (Mahler et al., 2017; Morrison et al., 2018).

Recent research in robotic waste sorting has increasingly moved from isolated classification models toward integrated perception–manipulation and planning frameworks. Recent reviews emphasize that practical robotic waste sorting requires the combined development of computer vision, AI-based recognition, gripper design, and robust manipulation strategies, while also identifying generalization, grasping robustness, and deployment cost as major remaining challenges (Satav et al., 2023). Kiyokawa et al. further highlighted that future robotic sorters for mixed industrial waste must handle not only object category recognition, but also object shape, pose, physical condition, and graspability under highly variable waste-stream conditions (Kiyokawa et al., 2022). In parallel, recent deep-learning studies have investigated real-time detection and classification methods for recyclable and household waste streams, while data-augmentation approaches such as WasteGAN have been proposed to reduce the dependence on large labelled datasets and improve robotic waste sorting in cluttered conveyor scenarios (Bacchin et al., 2024). These recent directions indicate a shift toward flexible, modular, and data-efficient waste-sorting systems, which is consistent with the small-batch collaborative workstation proposed in this paper.

Another recent work has also emphasized the importance of robot and application-agnostic planning frameworks for waste sorting (Kokkalis et al., 2025). It proposed a modular framework that decouples task-level scheduling from motion-level planning and supports flexible deployment across different robotic sorting platforms. While this approach addresses planning and scheduling challenges mainly in construction and demolition waste (CDW) sorting, the present work focuses on small-batch packaging waste sorting, where adaptability, reduced dataset dependency, and safe human–robot collaboration/interaction are the key design requirements.

A comparative overview of existing robotic waste sorting approaches presented in Table 1 shows existing systems predominantly target large-scale, high-throughput environments with low flexibility and high dataset dependency, whereas the proposed system is specifically designed for small-batch industrial scenarios requiring high adaptability and reconfigurability.

**TABLE 1:** Comparative overview of existing robotic waste sorting approaches.

Approach	Sensors/Input	Key methods	Advantages	Limitations	Application scale	Flexibility	Dataset dependency	Reconfig urability	Cost level
Vision-based robotic sorting (Lin et al., 2021)	RGB camera	CNN + object detection	High classification accuracy (>90%)	Requires large labelled datasets	Laboratory/pilot	Low	High	Low	Low
Deep learning robotic system (Kiyokawa et al., 2021)	RGB camera	CNN + pick-and-place	Good integration of perception and manipulation	Poor generalization to new objects	Small–medium scale	Medium	High	Low	Medium
Multi-sensor robotic sorting (Xiao et al., 2020)	Height map + NIR hyperspectral	Sensor fusion + ML	Improved material recognition	High system complexity	Industrial	Low	High	Low	High
Real-time robotic sorting (Boschetti et al., 2025)	RGB camera	Deep learning + control	Real-time processing	Limited adaptability	Industrial	Low	High	Low	High
AMP Robotics system (AMP Robotics, 2026)	RGB + proprietary sensors	AI-based recognition	High throughput	High cost, low flexibility	Large-scale industrial	Low	High	Very low	Very high
ZenRobotics Recycler (Lukka et al., 2014)	Multi-sensor	ML-based sorting	Proven industrial solution	Expensive, rigid setup	Large-scale industrial	Low	High	Very low	Very high
Dex-Net grasping (Mahler et al., 2017)	Depth/point cloud	Deep learning grasp planning	Robust grasping	Not waste-specific	General robotics	Medium	Medium	Medium	Medium
Real-time grasping system (Morrison et al., 2018)	RGB-D	Generative grasp synthesis	Works in clutter	Computationally demanding	Research	Medium	Medium	Medium	Medium
Mixed industrial waste robotic sorters survey (Kiyokawa et al., 2022)	Multi-sensor/vision-based system	Survey of robotic sorting, perception, grasping and system challenges	Comprehensive overview of mixed industrial waste sorting; identifies perception, grasping, object condition and system-integration challenges	Not a proposed workstation; no new experimental platform	Industrial/research	High	Medium	Medium	Not evaluated
RaapWaste robotic planning framework (Kokkalis et al., 2025)	RGB/hyperspectral camera; conveyor data	Robot-agnostic motion planning, task scheduling, ROS2/MoveIt2	Modular planning architecture; supports different robots and grippers; integrates simulation and real-world validation	Focused on CDW sorting; limited HRC integration; grasping depends on selected gripper and perception module	Industrial/research	High	Medium	High	Medium–High
Proposed Small-batch flexible station	RGB-D	SAM + DL + PointNet + DGCNN	High flexibility, modularity, HRC support, reduced dataset need	Computational complexity, grasp challenges	Small-scale industrial	High	Low–Medium	High	Medium

The main contributions of this study are:

- A hierarchical perception architecture for flexible small-batch waste sorting.
- A modular perception pipeline combining foundation models and deep learning for adaptive object segmentation with reduced dataset dependency.
- A modular robotic architecture enabling rapid reconfiguration in small-batch industrial environments.
- Laboratory validation of the proposed perception pipeline and demonstration of its integration within a collaborative robotic sorting workstation.

Together, these contributions establish a scalable and modular framework for small-batch waste sorting, addressing key limitations of existing systems related to dataset dependency, rigidity, and lack of flexibility in dynamic industrial environments. The present work should be viewed primarily as a system-level architectural and proof-of-feasibility study.

The remainder of the paper is organized as follows. Section 2 presents the system architecture of the small-batch flexible sorting station. Section 3 describes the methodology, including system integration, task scheduling, and manipulation strategy. Section 4 reports the laboratory validation and experimental results. Section 5 discusses the results, their implications, and the main limitations of the proposed approach. Section 6 addresses industrial relevance and economic impact, while Section 7 concludes the paper and outlines directions for future work.

## 2 System architecture

A typical robotic waste-sorting system, illustrated in Figure 1A, consists of a pick-and-place solution operating above a conveyor belt. The robot is controlled by a computer vision system comprising one or more cameras and an algorithm designed to interpret image content, determine object position, and execute the grasping task. In this context, a key factor for overall system efficiency is the capability of computer vision algorithms to recognize different types of waste, as well as the robustness of the grippers required to handle and relocate highly variable objects.

**FIGURE 1:**
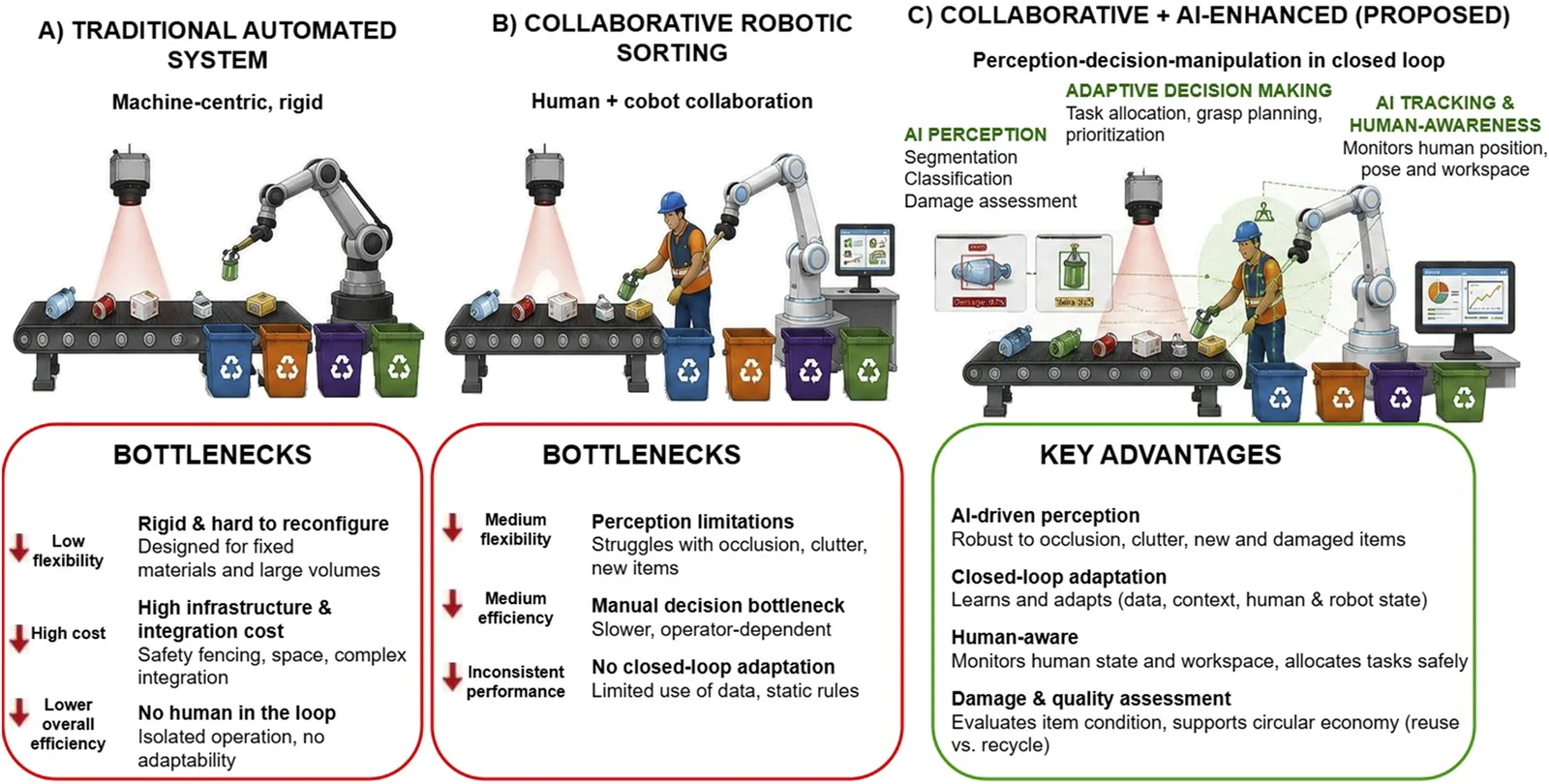
**(A)** Illustration of the key elements of the packaging waste sorting workstation, consisting of a computer vision system, conveyor belt and robot; **(B)** Illustration of the key elements of the packaging waste sorting workstation for a safe human-robot-collaboration, consisting of a computer vision system, conveyor belt, cobots and human operator. **(C)** Illustration of workstation with cobot and human operator AI empowered with proposed approach.

The main components of the robotic waste-sorting system shown in Figure 1A are: 1) Vision system–camera-based system used for object detection and data acquisition; 2) Gripper–end-effector mounted on the robot for grasping and manipulating waste items; 3) Conveyor belt–transports waste items through the detection and sorting zone; 4) Computer–processes visual data, runs artificial intelligence algorithms for classification, and controls the robotic system; and 5) Robot–performs pick-and-place operations based on classification results.

The small-batch waste-sorting system (illustrated in Figure 1B) is based on the same core components described above: a vision system, gripper, conveyor belt, computer, and robotic manipulator. However, in contrast to the conventional configuration shown in Figure 1A, the robot is replaced with a collaborative robot (cobot), enabling safe human–robot collaboration (HRC) in small-batch sorting environments. In addition, the system includes human operator(s) as the sixth component, responsible for performing complementary sorting tasks and supporting the robotic system (i.e., remove obstacles for vision system). The final stage of proposed small-batch waste-sorting system (illustrated in Figure 1C) is AI enhanced version of system from Figure 1B with solutions proposed in this paper.

The primary objective of the proposed system is the efficient and safe sorting of waste items into designated bins. The system is controlled by a centralized task scheduler that continuously loops to evaluate and assign tasks based on real-time sensory input. The integration of these components enables automated object detection, classification, and manipulation in a flexible, modular, and scalable waste-sorting solution aligned with circular economy and industry 5.0 principles. The collaborative Small Batch Flexible Station is designed to sort selected types of waste by detecting objects from waste stream on a conveyor belt using a vision system (camera), classifying them through an artificial intelligence algorithm, and performing a “pick-and-place” operation (cobot with gripper) to place the objects into predefined bins locations. Systems designed with cobots provide a safer working environment compared to traditional rigid industrial robots, as they are intended for safe human-robot interaction and operation in shared workspaces.

### 2.1 Hardware architecture

Small-batch sorting system uses following key components:

1. Cobot: MAiRA Pro M (Neura Robotics, 2025) is 7DoF cobot equipped with torque sensors in each joint and 6 axis F/T sensor in end-effector. This makes MAiRA Pro M suitable for dynamic environment waste-sorting applications due to its combination of precision, advanced sensor integration, and built-in safety features, providing a safe physical HRC. Although MAiRA Pro M was selected for the experimental realization, the proposed system architecture is not dependent on this specific cobot model. Other collaborative robots can be integrated, provided that they offer suitable performances (i.e., payload, workspace, safety functionality, ROS-based control architecture).
2. Vision system: Intel RealSense D455 stereo depth camera (Intel Corporation, 2020) was used as vision system. Camera has 10 m depth range with 2% precision at 4 m, depth resolution up to 1,280 × 800 at 30 fps, enabling detailed 3D reconstruction of waste items, including shape and volume estimation.
3. Gripper: For the end-effector, a modular soft gripper from the SoftGripping construction kit (SoftGripping GmbH, 2024) was integrated, precisely its four-finger configuration. The choice of grippers is large and depends on the shape and dimensions of the object being picked. Therefore, gripper was selected based on best results for grasping of typical packaging waste items (Kiyokawa et al., 2022) and results of previous grasping testing (Klasanović et al., 2024). This gripper is particularly suitable for packaging waste sorting due to its capability for gentle handling of deformable objects, modularity, and adaptability to irregularly shaped items. Silicon made flexible fingers adapt to a wide range of object geometries, enabling secure and damage-free grasping of fragile components such as cans, Tetra Pak, and glass or plastic bottles. Modular design allows easy adjustment of the number of fingers (2–6), spacing, and mounting interfaces, supporting rapid reconfiguration for different sorting tasks. Soft gripper is a versatile and safe tool for handling the diverse and unpredictable mix of objects found in waste streams.

The cobot, gripper, and vision system are the key elements of the waste sorting system and primarily determine the system’s capability for efficient waste sorting. Additional system components that are necessary for workstation operation include:

- Conveyor belt for guiding the waste flow. To maintain a constant conveyor speed, actuators (electric motors) and a frequency inverter (variable frequency drive) are required.
- Compressor and pneumatic control unit for operating soft grippers or vacuum grippers.
- Monitoring computer for process supervision and control. The computer runs vision system and robot control algorithms, while providing visual feedback on how accurately the algorithm detects objects on the conveyor, how effectively the robot tracks them, and how successfully the task is executed.

### 2.2 Vision system software architecture

In the proposed waste sorting system, waste items are transported through a shared manipulation area where a collaborative robot and a human operator concurrently perform sorting actions. Under these conditions, the perception stack must (i) localize each item in the conveyor reference frame, (ii) provide a waste category label that is consistent with the downstream material stream (e.g., cans, bottles), and (iii) extract geometrical information needed for robust grasp synthesis and routing decisions.

To address these requirements, the perception stack is designed as a hierarchical and modular architecture.

The perception stack is organized into three functional levels:

- The first level provides core object localization and classification required for real-time operation.
- The second level introduces geometric reasoning through point-cloud-based shape recognition, supporting robust grasp planning.
- The third level extends the system with damage assessment capabilities, enabling safety-aware decision-making and task allocation in human–robot collaboration.

At the system level, RGB and depth streams are aligned using the Intel RealSense SDK. Intrinsic parameters are retained to enable metric reconstruction in the camera frame. The perception module exposes a unified interface consisting of segmentation masks, bounding boxes, category labels, confidence values, and 3D descriptors, enabling seamless integration with grasp planning, safety monitoring, and task scheduling components.

The aligned RGB–D data is processed using instance-level segmentation to isolate individual waste items from the conveyor and surrounding structures. Segmentation masks are used to compute 2D object descriptors (bounding boxes, contours, confidence values) and to restrict depth-to-point-cloud conversion to object regions, thereby suppressing background and improving geometric stability.

The modular structure of the perception stack is intentionally aligned with the system-level architecture of the collaborative workstation: object recognition provides the minimum information needed to trigger a pick-and-place cycle, while optional branches (damage assessment and shape reasoning) enrich the decision layer whenever higher-level sorting logic is required.

#### 2.2.1 Perception module (localization and classification)

The goal of this module is to isolate individual waste items from the conveyor belt and surrounding structures from the RGB–D data stream. For these purposes, we utilised the procedure proposed in our previous work (Vukicevic et al., 2025), which fuses the Segment Anything Model (SAM) (Kirillov et al., 2023) with classification (MobileNetV2) architecture (Sandler et al., 2018). This approach enables flexible adaptation to diverse waste streams and small-batch industrial scenarios without requiring extensive retraining and data-annotation effort (particularly data annotation, since the data preparation is reduced from instance segmentation to the classification of cropped objects into corresponding class-folders). Since the output may result with several composing segmentation proposals within one physical object (Figure 2), a post-processing step is applied to merge object parts. Objects that are almost completely enclosed within a larger object-level bounding box are treated as object subregions (Figure 2C, object 4 is within object 5), while for the rest we checked neighbouring objects-masks, and merged them if they share more than 20% of the joint contour (e.g., Figure 2C, objects 1 and 2 will be merged, and after that the resulting object will be merged with the object 3 etc.). After the merging, the resulting object class is assigned by using the pixel counting to find the most dominant material (each composing part is previously classified using the MobileNetV2). This ensures that each waste item is represented by one bounding box and mask before being passed to the downstream modules to trigger pick-and-place operations.

**FIGURE 2:**
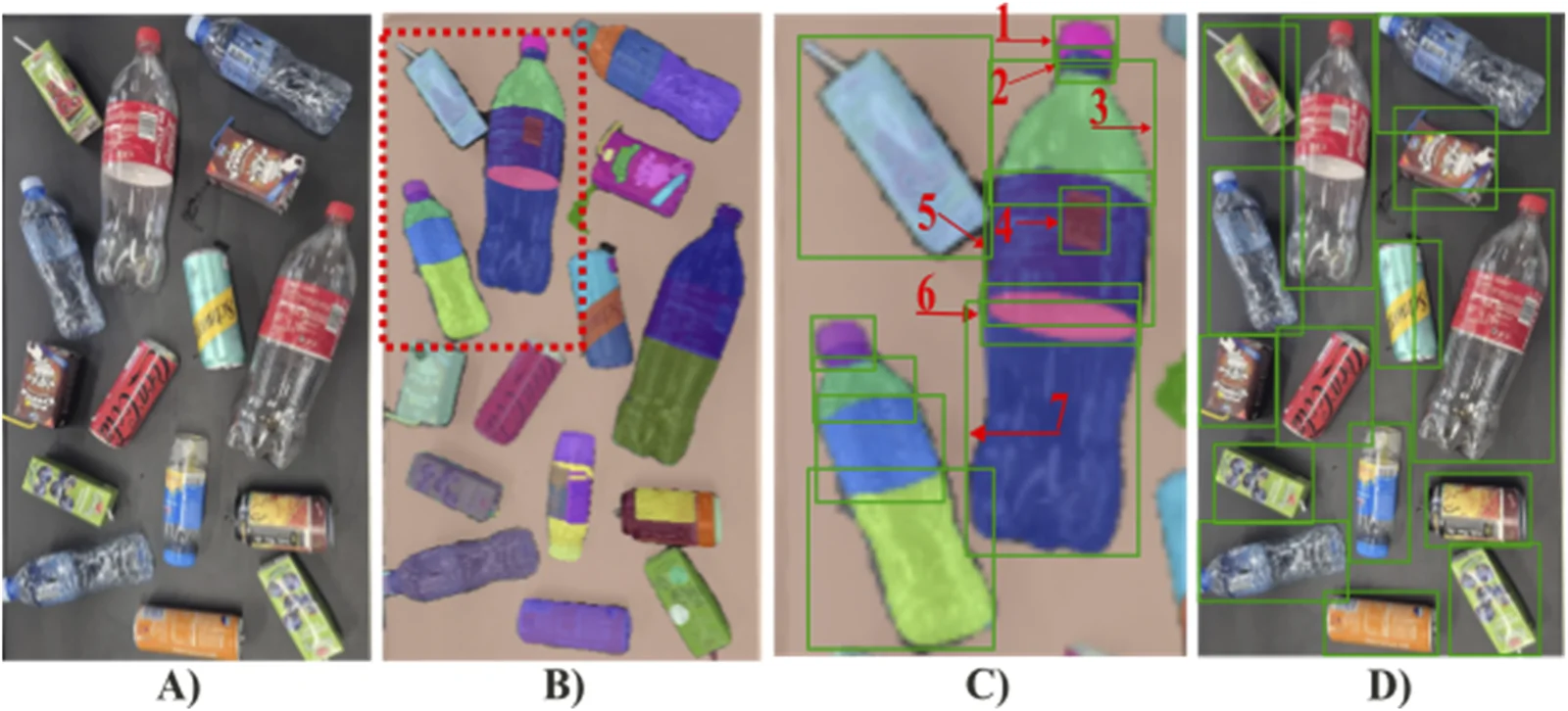
Example of nested-detection filtering in the SAM-based perception pipeline: **(A)** Raw RGB image of packaging waste items on the conveyor belt. **(B)** Initial SAM segmentation output, where multiple segmentation proposals may be generated for the same physical object. **(C)** Detailed example of nested detections, where smaller bounding boxes correspond to object parts such as labels or local surface regions rather than separate physical items. **(D)** Final post-processed detections after filtering nested bounding boxes, retaining one dominant bounding box per physical object. The resulting detections are used for downstream classification, RGB-D localization, and robotic grasp planning.

The model was trained on a dataset composed of two public sources: the Kaggle Waste Materials Classification Dataset (Ritharson, 2023) and the ANTLab (Politecnico di Milano) Smart Waste Bin Dataset (Longo et al., 2021). From the Kaggle dataset, 1,987 images were selected, while 4,600 images were randomly sampled from the ANTLab dataset (out of 5,199 total images), covering tin cans, Tetra Pak, and plastic bottles. For classifier training, the dataset was randomly split into training, validation, and test subsets using a 70%/15%/15% split. During training, online augmentation was applied with a probability of 20%, including random rotation within ±30°, random flipping, random cropping, and Gaussian noise. Across the evaluated datasets, performances were in the range of approximately 86%–97%, with reported performance of 0.94 accuracy on packaging-relevant waste streams.

#### 2.2.2 Geometric reasoning module (grasping planning)

To improve robustness of grasping under conditions of occlusion, contamination, and visual ambiguity, we incorporate a complementary geometric shape reasoning based on 3D point-cloud processing. Point-cloud processing is performed using Open3D and includes filtering, clustering, normalization, and downsampling to ensure stable and consistent input representation. Selection of grasping strategy is done by using the PointNet architecture (Qi et al., 2017), which processes unordered point sets and extracts global geometric features. The network uses transformation networks (T-Nets) to achieve invariance to translation and rotation, followed by point-wise feature extraction and global max-pooling. The module assigns each object to a grasp-relevant geometric primitive (e.g., cylindrical, box-like, irregular), enabling:

- Robust grasp strategy selection,
- Operation under partial occlusion,
- Independence from texture and visual appearance.

Despite being trained on idealized primitives, the shape classifier generalizes to practical waste items by assigning them to the closest visually similar class, effectively yielding a grasp-relevant abstraction. In the packaging waste context, this allows the robot controller to associate bottles and cans with a cylinder-like category and Tetra Pak with a rectangular prism category, and deformable packaging with intermediate geometries. The resulting geometric label can be used as an input feature in grasp planning and in the decision logic that coordinates robot and human actions. Moreover, because the module operates on geometry, it is largely invariant to surface textures and printing, which can vary dramatically across packaging brands. As a result, shape-based recognition serves as a robust secondary cue that complements appearance-driven material classification within a collaborative sorting workflow. The outputs of this module include: geometric class label, and shape descriptors for grasp planning.

#### 2.2.3 Damage assessment module

Packaging waste sorting in circular economy applications requires not only material classification but also assessment of object condition, as it directly influences manipulation strategy and safety in human–robot collaboration. Damage information serves as a decision variable for both routing and manipulation: heavily deformed items may require adapted grasping strategies, while sharp or broken objects should be preferentially handled by the robot to reduce risks for human operators. Consequently, damage assessment enables safety-aware task allocation, adaptive grasp planning, and prioritization of hazardous objects for robotic handling.

The damage estimation module operates on synchronized RGB–D data and follows a multi-stage pipeline as illustrated in Figure 3. The processing pipeline includes depth filtering, clustering, normalization, and fixed-size sampling to ensure robustness and computational efficiency. The Dynamic Graph Convolutional Neural Networks (DGCNN) (Wang et al., 2019) model captures deformation-sensitive features, while the fuzzy estimator refines classification outputs into a continuous damage metric using calibrated parameters. As a result, each object is assigned: a discrete damage class (intact, damaged, destroyed), and a continuous damage score (0%–100%). The model is trained using the Adam optimizer with early stopping (Kingma and Ba, 2014), ensuring stable convergence and generalization.

**FIGURE 3:**
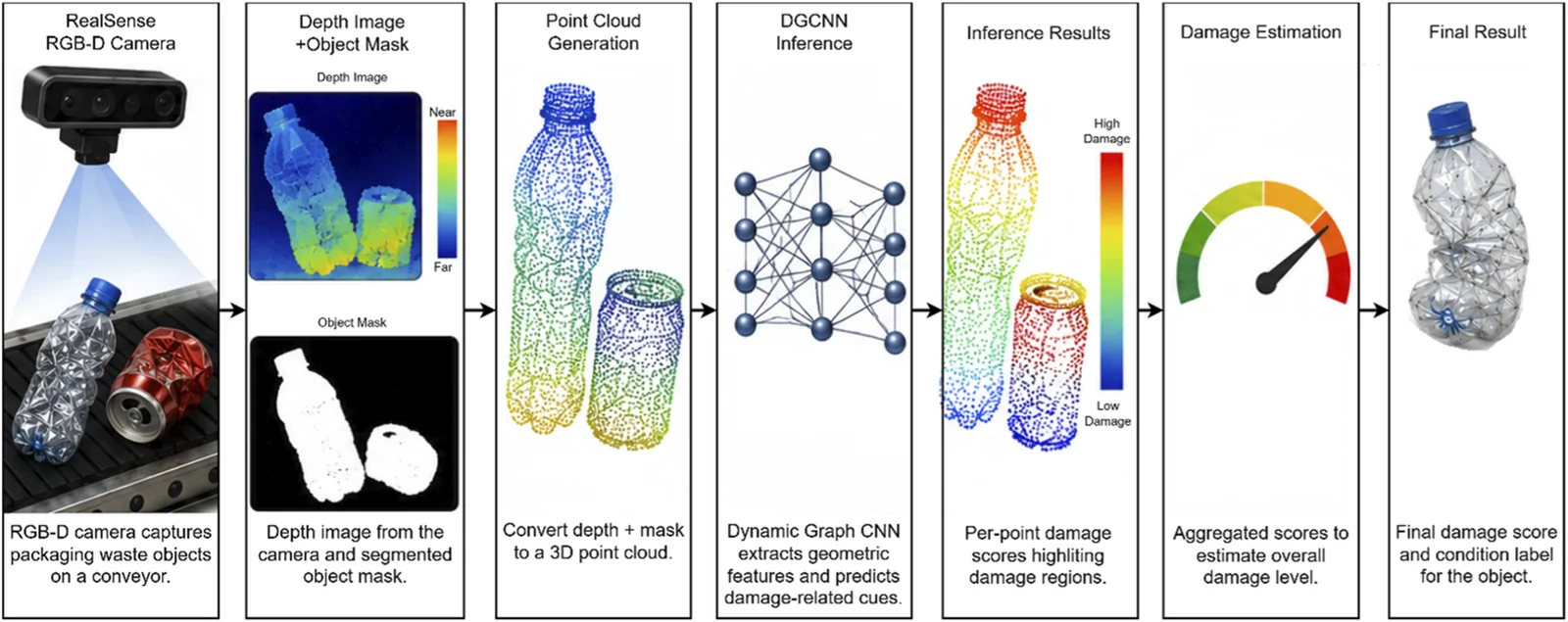
Illustration of end-to-end pipeline of the proposed RGB–D-based damage estimation module.

## 3 Methodology

This section describes the methodological basis for integrating the proposed perception, decision-making, and manipulation components into a collaborative waste-sorting workstation. The focus is placed on how functional outputs from the vision modules are transformed into task-allocation and robot-control inputs, and how the selected gripper configuration supports reliable manipulation of packaging waste. The methodology therefore covers both the system-level integration logic and the manipulation-subsystem evaluation used to justify the end-effector selection.

### 3.1 System integration and functional outputs

The perception modules were designed as interoperable components of a collaborative workstation rather than stand-alone demonstrators. The vision subsystem establishes a closed perception–action loop with the robot controller: segmentation extracts object pose and geometry, material recognition determines the destination bin, while optional quality descriptors (damage score and geometric class) further refine routing and manipulation. This layered architecture supports multiple operating modes. A basic configuration provides object localization and classification sufficient for conventional pick-and-place operations, whereas advanced configurations additionally estimate damage and geometric primitives to optimize grasp approach vectors, gripping forces, and ballistic placement trajectories.

Explicit uncertainty estimation is essential for reliable human–robot collaboration. Occluded, overlapping, or out-of-distribution objects are labelled as unknown, allowing safe delegation to the human operator instead of executing low-confidence grasps. Conversely, highly damaged objects can be preferentially assigned to robotic handling, reducing operator exposure to sharp edges and fractured surfaces, while intact items can be directed toward reuse-oriented processing. Although this decision framework supports collaborative task scheduling on moving conveyors, its quantitative evaluation remains future work.

The computational characteristics of the perception stack support deployment in industrial environments. Because the framework relies on modular software components, including Open3D, and the RealSense SDK, new packaging types, damage categories, and site-specific imaging conditions can be accommodated through retraining without modifying the overall system architecture. This adaptability is particularly important in circular-economy applications, where packaging designs and material streams continuously evolve.

Beyond real-time sorting, the perception modules generate structured information that can be stored together with timestamps and destination bins, enabling performance monitoring, traceability, and downstream analytics. These data also facilitate integration with digital twins and cloud-based management services, such as reference-geometry retrieval or inspection reporting. Consequently, the vision system contributes not only to robotic manipulation but also to the broader objectives of collaborative sorting by improving operational efficiency, worker safety, and decision support for circular-economy applications.

The proposed workstation is intended to operate downstream of a conventional industrial feeding stage that regulates material flow before objects enter the perception zone. Rather than processing completely random bulk material, packaging waste is mechanically preconditioned using industrial feeding equipment such as vibrating feeders, metering conveyors, guiding rails, passive spreaders, rotating drums, or mechanical separators. These upstream processes reduce excessive stacking and promote object singulation, thereby improving the reliability of segmentation, pose estimation, and robotic manipulation. Consequently, mechanical singulation is considered an external support process rather than a component of the proposed perception pipeline, reflecting the intended deployment of the workstation in controlled small-batch industrial packaging waste scenarios.

### 3.2 Waste sorting station architecture

The proposed Waste Sorting Station is implemented as a ROS (Robot Operating System) architecture (Open Robotics, 2025) integrating the collaborative robot, conveyor system, vision sensors, and human-monitoring modules. Robot motion planning is performed using the MoveIt package (MoveIt, 2026), while machine-learning algorithms provide object detection and classification. All subsystems communicate through a central ROS node that coordinates synchronization, safety, and real-time execution.

The system architecture (Figure 4) employs a data-driven task scheduler that distributes sorting tasks between the robot and the human operator. As waste items enter the camera field of view, the perception system extracts five parameters required for task allocation:

- Waste classification,
- Object position,
- Damage level,
- Safety level,
- Conveyor speed.

**FIGURE 4:**
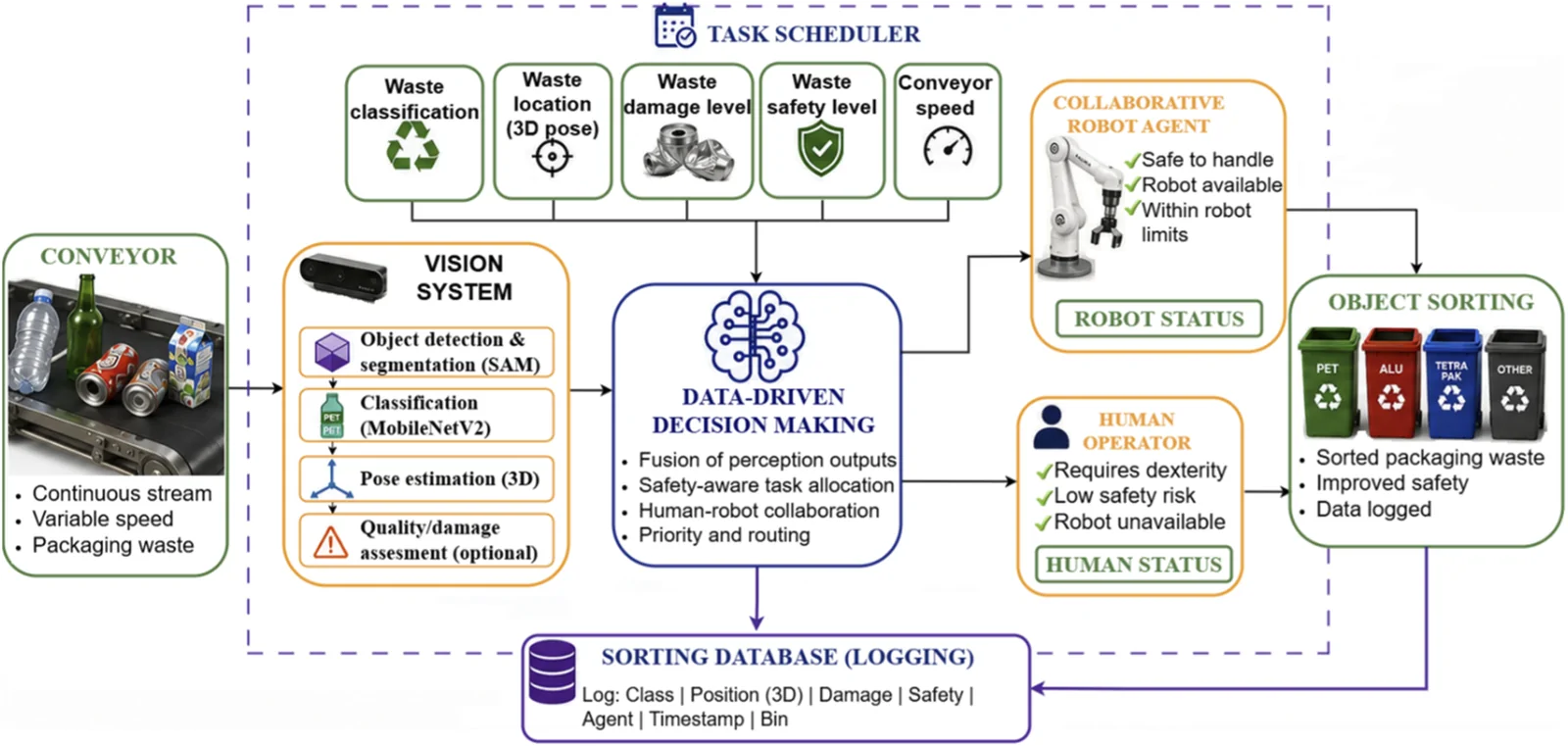
Block structure of task scheduler system architecture.

The Task Scheduler combines these perception outputs with the real-time availability of both agents. Objects satisfying predefined safety and manipulation constraints are assigned to the robot, whereas objects requiring human dexterity or exceeding the robot’s safe handling capabilities are delegated to the operator. All completed sorting actions are subsequently recorded in the system database.

The proposed architecture follows the hierarchical scheduling paradigm presented in (Kokkalis et al., 2025), where task allocation is separated from motion planning to improve modularity and hardware independence. In contrast to previous approaches, the proposed scheduler additionally incorporates human availability together with object safety and damage information during task assignment. The scheduler presented in this work represents a proof-of-concept implementation, while quantitative evaluation of task allocation efficiency and operator intervention frequency remains future work.

### 3.3 Software architecture

The software architecture is based on a distributed ROS2 framework that provides low-latency communication among all hardware components. A dedicated camera node performs object detection and classification, continuously publishing object coordinates and physical attributes. The Task Scheduler interfaces with the NEURA_SDK to convert high-level motion commands into real-time robot control signals. Conveyor synchronization is achieved through the industrial OPC UA protocol, enabling the ROS2 environment to access encoder data and continuously update the predicted rendezvous point for dynamic grasping. The overall system workflow is summarized by the pseudocode presented in Figure 5.

**FIGURE 5:**
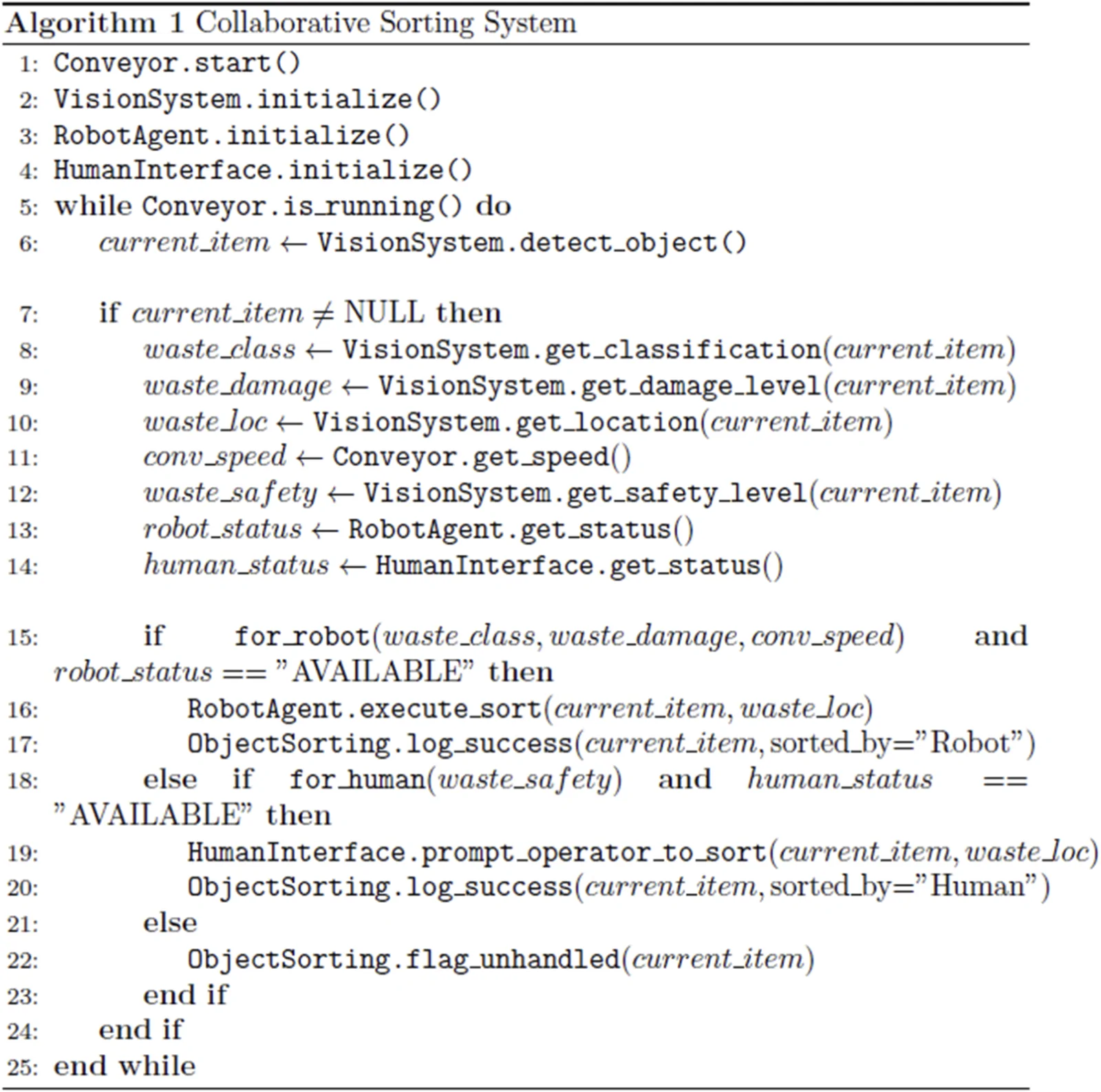
Pseudo code for illustrating the logic of task execution for Collaborative sorting work cell.

### 3.4 Manipulation and gripper selection

Reliable manipulation is essential for automated packaging waste sorting because object geometry, deformation, and mass distribution directly affect grasp stability. Based on our previous experimental evaluation of soft pneumatic grippers (Klasanović et al., 2024), the proposed workstation employs a four-finger soft pneumatic gripper, as this configuration demonstrated the greatest robustness under representative manipulation uncertainties encountered in conveyor-based packaging waste sorting. Grasp quality was assessed using a seven-level normalized performance metric ranging from failed grasping (0) to fully stable grasping under perturbations (1). The three-finger gripper achieved the highest average performance under nominal conditions (0.86), whereas the four-finger configuration demonstrated superior robustness under uncertainties in object position, orientation, and mass distribution, achieving the highest scores in all robustness-related experiments. Because packaging waste on moving conveyors exhibits significant variability in pose, deformation, and mass distribution, the four-finger configuration was selected for the proposed workstation.

## 4 Experimental results

During laboratory validation, industrial feeding was emulated by manually placing packaging waste items on the conveyor with sufficient spacing between consecutive objects. Under these controlled conditions, the workstation demonstrated stable perception, RGB-D localization, conveyor tracking, coordinate transformation, and conveyor-based pick-and-place execution.

The laboratory setup is shown in Figure 6. The network was trained to detect and segment three most common recyclable packaging classes that are representative for pilot deployments in sorting lines: aluminium cans, plastic bottles, and Tetra Pak. Objects representing the waste stream are placed on the conveyor belt. During laboratory experiments, objects were manually introduced onto the conveyor belt in a spaced configuration, emulating the output of an upstream industrial feeding or singulation mechanism. The purpose of this arrangement was to obtain repeatable experimental conditions for validating the perception–localization–manipulation loop. Therefore, the examples shown in Figure 6 correspond to separated packaging items rather than dense cluttered piles. This assumption is consistent with the intended small-batch industrial use case, where basic mechanical preconditioning can be applied before the robotic sorting workstation. These objects are identified using the Intel RealSense D455 depth camera.

**FIGURE 6:**
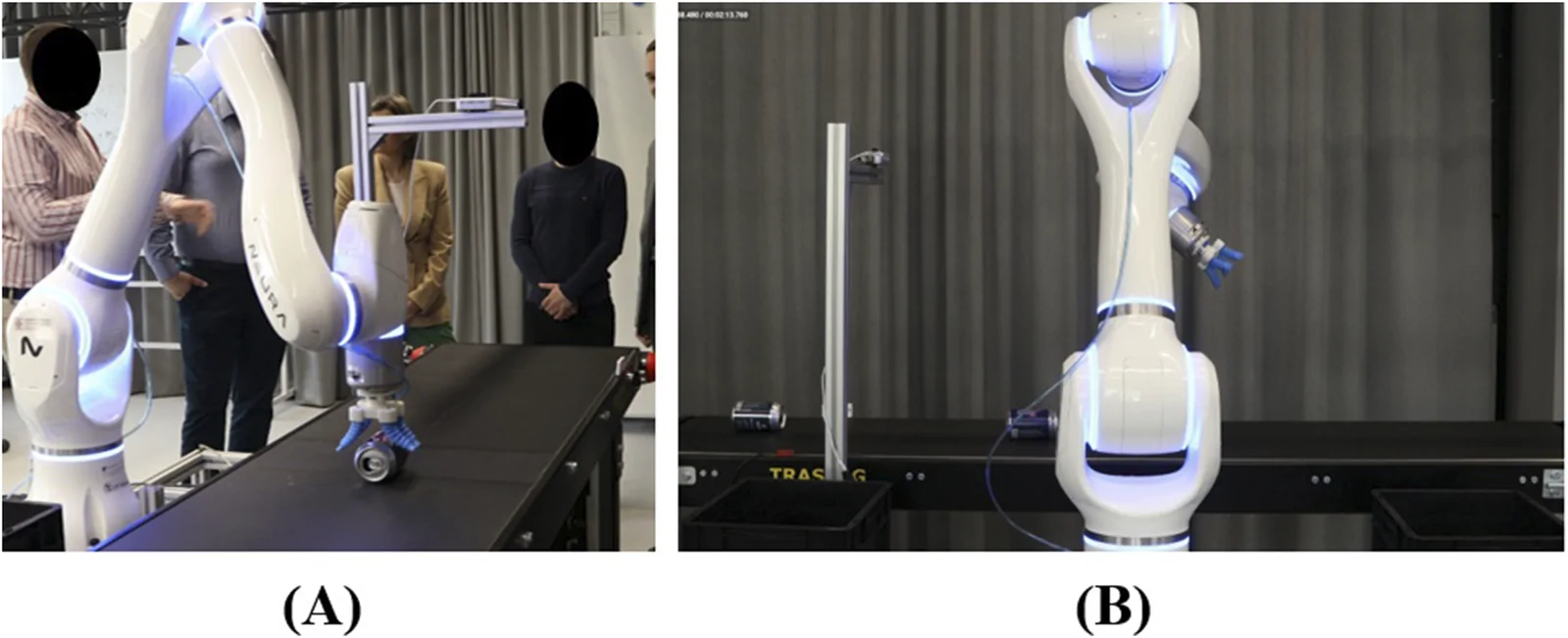
Laboratory setup of waste sorting station. **(A)** Side view; **(B)** back view (right).

Based on the position of the robot’s end-effector and the detected object location, the system calculates the appropriate picking position and generates the corresponding motion trajectory for object grasping and sorting.

The metrics selected for the evaluation of the proposed Core Perception Pipeline included:

$$Accuracy=\frac{T_{p}+T_{n}}{T_{p}+F_{p}+T_{n}+F_{n}},Precision=\frac{T_{p}}{T_{p}+F_{p}},$$

$$Recal=\frac{T_{p}}{T_{p}+F_{n}},F1-score=2\frac{Recall*Precision}{Recall+Precision},$$

where $T_{p}$ , $T_{n}$ , $F_{p}$ and $F_{n}$ are true positive, true negative, false positive, and false negative classifications, respectively. The obtained metric values are summarized in Table 2.

**TABLE 2:** Classification performance of the developed SAM + MobileNetV2 perception pipeline applied to packaging waste.

Metrics	Accuracy	Precision	Recall	F1-score
Value	0.93	0.93	0.92	0.92

The objective of Table 2 is to verify the suitability of the selected perception pipeline for integration into the proposed workstation. Comparative evaluation of alternative SAM variants and classification architectures has been reported in (Vukicevic et al., 2025). The obtained results show high performance across all evaluated metrics. To further improve computational efficiency and reduce inference time, FastSAM and EfficientSAM variants are recommended as faster alternatives to the baseline SAM model (Vukicevic et al., 2025).

Computational performance was also evaluated for the complete perception pipeline on a workstation equipped with an NVIDIA TITAN RTX GPU with 24 GB of VRAM. Under the configuration used in the experiments, the average processing time was 238.1 ms per frame, corresponding to an effective processing rate of approximately 4.2 FPS. Depending on the enabled modules and selected processing settings, the execution time may increase to approximately 1–2 s per frame.

SAM was retained in the present implementation for practical reasons, as it had already been integrated and validated within the complete perception–localization–manipulation workflow. The objective of this study was system-level proof-of-feasibility rather than optimization of segmentation latency. For applications requiring higher processing rates, the modular architecture permits replacement of the baseline SAM implementation with more computationally efficient variants, such as FastSAM or EfficientSAM, without changing the remaining system architecture. Accordingly, the manuscript does not claim strict real-time operation of the complete perception pipeline.

In addition to the SAM + MobileNetV2 pipeline, the DGCNN-based damage assessment module was quantitatively evaluated on object-specific validation datasets. The achieved validation accuracies were 83.5% for aluminium cans, 88.2% for plastic bottles, and 78.7% for Tetra Pak packages, corresponding to an overall validation accuracy of 83.9% across 740 validation samples. Validation accuracies data is given in Table 3. The continuous damage score (0%–100%) is obtained by calibrating the class probabilities of the three discrete damage levels (0%, 50%, and 100%) rather than by direct regression. The PointNet-based geometric reasoning module is included as an optional component of the perception architecture and is illustrated qualitatively in this study; its standalone statistical evaluation is left for future work.

**TABLE 3:** Validation performance of the DGCNN damage-assessment module.

Object	Aluminium cans	Plastic bottles	Tetra pak	Overall
Validation samples	236	288	216	740
Accuracy	83.5%	88.2%	78.7%	83.9%

Figure 2 illustrates the effect of nested-box filtering on the SAM segmentation output. Redundant internal proposals corresponding to labels, caps, or local surface regions are removed, leaving one representative bounding box per physical object for classification, localization, and grasp planning. This example demonstrates redundancy removal under controlled conveyor conditions and should not be interpreted as validation under highly cluttered municipal waste streams.

Figure 7 presents representative outputs of the integrated perception pipeline after nested-box filtering. The remaining object-level detections are classified and combined with RGB-D information to estimate approximate 3D object locations. These outputs provide the minimum information required for downstream task scheduling and robot control, including object category, bounding box, confidence score, and spatial position. The examples show that the proposed pipeline generates stable object-level detections for the selected packaging waste categories under controlled conveyor conditions.

**FIGURE 7:**
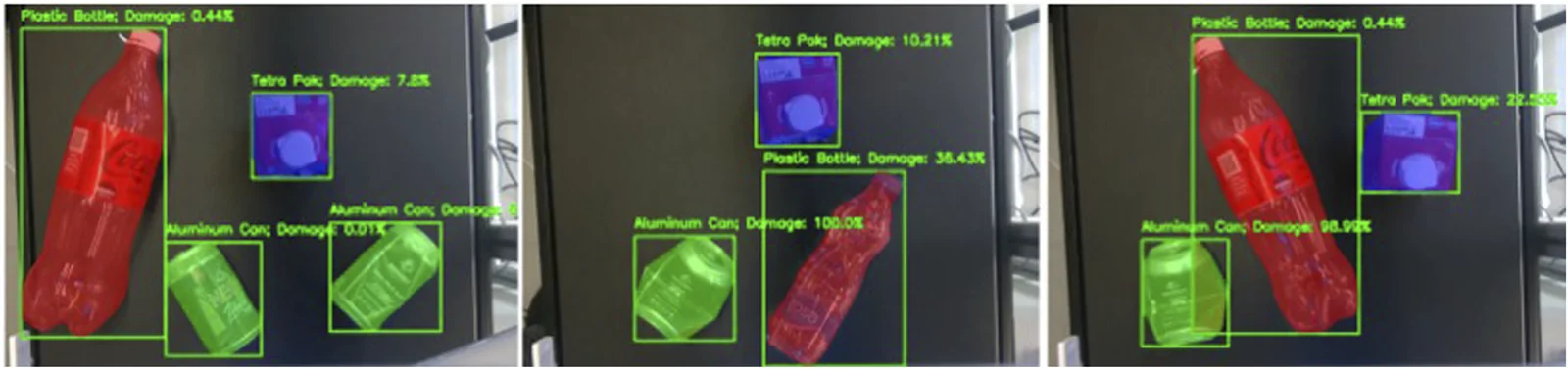
Examples of detection and classification of objects from waste stream: bottles, tin cans and Tetra Pak, along with damage estimation - outputs from the damage-assessment module.

In addition to material classification, the system provides auxiliary semantic information relevant to robotic manipulation, including geometric descriptors and damage-related cues. These outputs support grasp-oriented reasoning and safety-aware task allocation by distinguishing between intact objects, deformed items, and objects that may require preferential robotic handling. Therefore, Figure 7 demonstrates not only visual recognition, but also the generation of structured perception outputs required for collaborative waste-sorting operation.

The proposed six-phase pick-and-place procedure is consistent with recent planning-oriented waste-sorting approaches, where grasp execution on a moving conveyor requires reachability checking, approach motion, synchronized tracking, object lifting, and transfer to the target bin. Compared with generic planning frameworks such as RaapWaste (Kokkalis et al., 2025), the present implementation is tailored to a collaborative packaging-waste workstation and explicitly links the motion sequence with safety-aware task allocation between the cobot and the human operator.

For items assigned to the collaborative robot agent, the system executes a dynamic pick-and-place operation without halting the conveyor. This is managed by a discrete, six-phase state machine algorithm designed to synchronize the robot’s end-effector with the moving target. The phases are executed sequentially as follows:

- Phase 1 (Intersection): The robot initiates motion toward an estimated pick point where the end-effector and object trajectories will intersect, calculated using the object’s initial location and the conveyor velocity (Figure 8A).
- Phase 2 (Alignment): The robot reaches the alignment position, synchronizing gripper velocity with velocity of the object along the with conveyor line to ensure the end-effector and object positions overlap precisely (Figure 8B).
- Phase 3 (Grasping): While actively tracking the object’s movement on the conveyor, the robot actuates the end-effector to grasp the object (Figure 8C).
- Phase 4 (Lifting): The robot retracts vertically to lift the object, safely clearing the moving conveyor belt (Figure 8D).
- Phase 5 (Moving): The robot breaks synchronization with the conveyor and moves above the designated sorting bin determined by the initial waste classification (Figure 8E).
- Phase 6 (Releasing): The end-effector opens, releasing the object into the bin, after which the robot returns to its idle home position to await the next assignment (Figure 8F).

**FIGURE 8:**
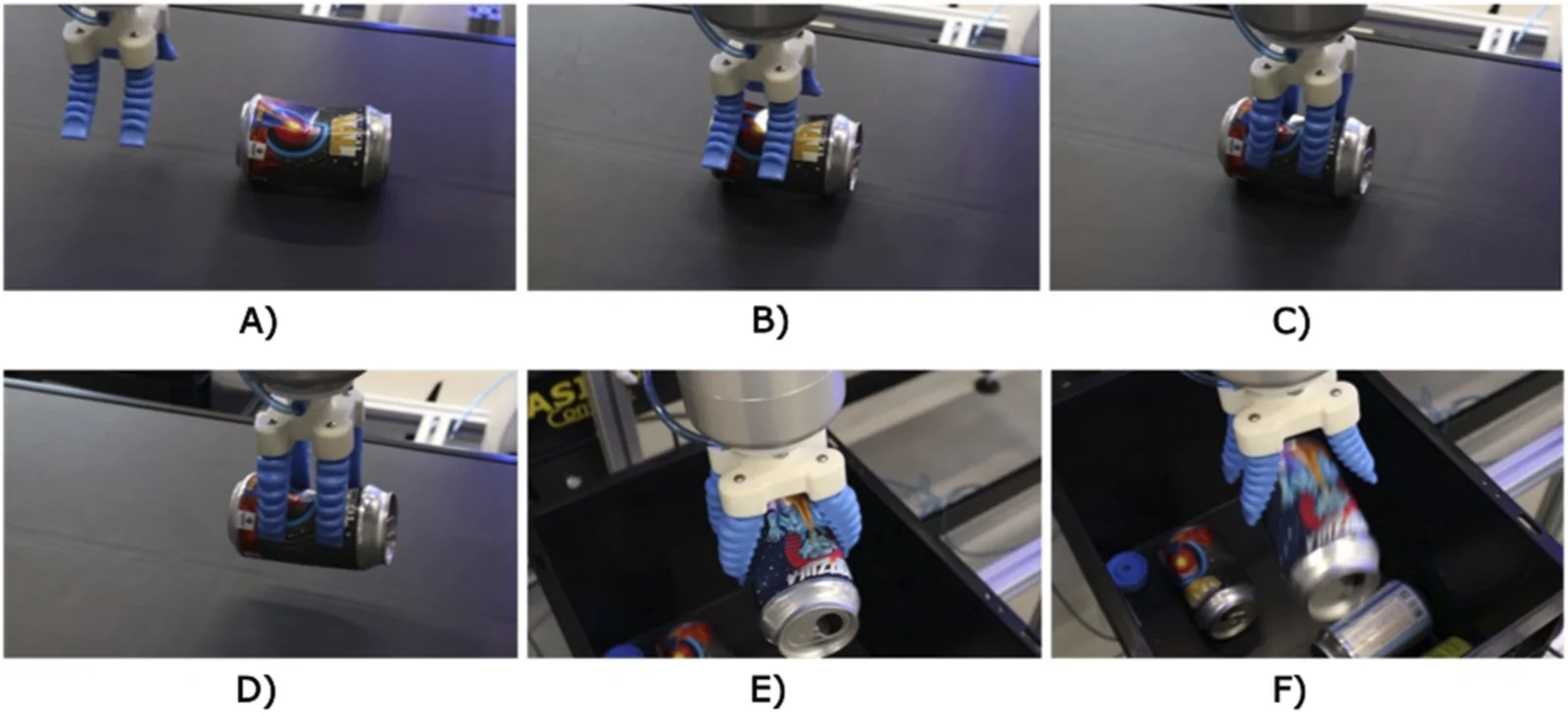
Illustration of six phases in the end-effector trajectory during object grasping: **(A)** Phase 1 (Intersection) - The robot initiates motion toward an estimated pick point; **(B)** Phase 2 (Alignment) - Reaching the alignment position, where the end-effector and object positions overlap and velocity align; **(C)** Phase 3 (Grasping) - Grasping the object; **(D)** Phase 4 (Lifting) - Lifting the object; **(E)** Phase 5 (Moving) - Moving above the designated sorting bin; **(F)** Phase 6 (Releasing) - Releasing the object into the bin.

The accompanying pseudo code is provided in Figure 9.

**FIGURE 9:**
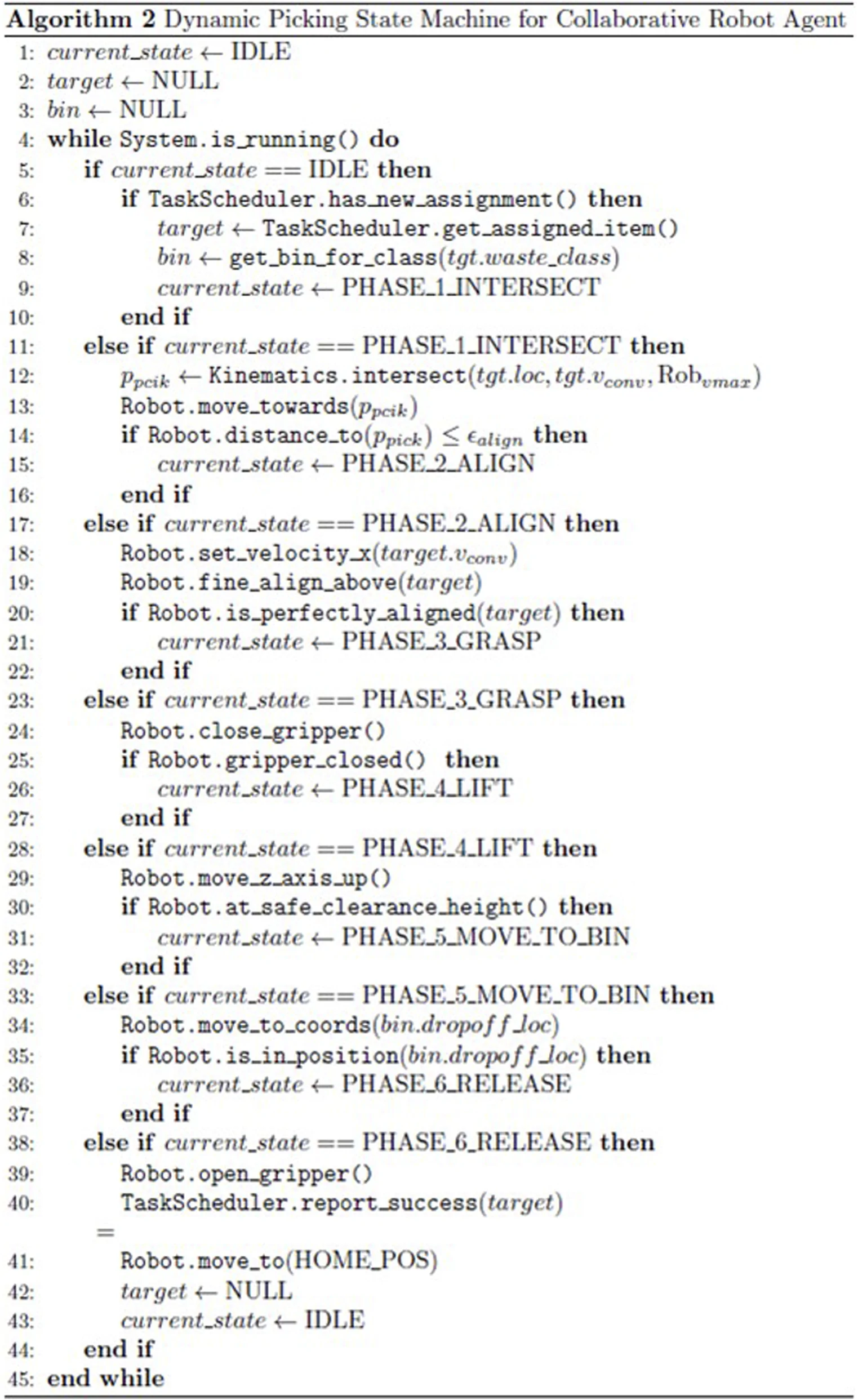
Pseudo code for implementing the 6-phase pick and place algorithm.

Table 4 summarizes the manipulation and throughput performance of the proposed workstation under controlled conveyor-based sorting conditions. Out of 200 grasp attempts, 184 were successful, corresponding to a grasp success rate of 92.0%. Among the successfully grasped objects, 178 completed the full six-phase pick-and-place sequence, resulting in a pick-and-place success rate of 96.7% for grasped objects and an overall end-to-end sorting success rate of 89.0%. The mean cycle time was 10.47 s per object, corresponding to an estimated sorting rate of 5.73 picks/min or 343.84 picks/h.

**TABLE 4:** Manipulation and throughput performance of the workstation under controlled conveyor-based sorting conditions.

Metric	Value	Trials/basis
Grasp success rate	92.0%	184/200
Pick-and-place success rate for grasped objects	96.7%	178/184
End-to-end sorting success rate	89.0%	178/200
Mean cycle time	10.47 s/object	200 cycles
Sorting rate	5.73 picks/min 343.84 picks/h	Derived from cycle time

An analysis of the 16 failed grasp attempts identified three failure modes: failure to establish a stable initial grasp due to severe object deformation in 7 cases, object loss during transfer to the target bin in 5 cases, and positional or angular misalignment between the gripper and the object in 4 cases. These results indicate that the main remaining limitations are related to grasp stability during robot motion, highly deformed object geometries, and the accuracy of gripper-to-object alignment.

## 5 Discussion

### 5.1 Interpretation of perception performance

The experimental results demonstrate that the proposed perception pipeline provides reliable object recognition for collaborative packaging waste sorting while maintaining the flexibility required for small-batch industrial applications. The achieved classification performance (Table 2), together with the qualitative results presented in Figures 6, 7, indicates that the combination of foundation-model-based segmentation and lightweight image classification can successfully support downstream robotic manipulation without requiring task-specific segmentation training.

An important observation is that perception accuracy alone does not determine the performance of an autonomous sorting system. In robotic manipulation, perception errors propagate directly to localization, grasp planning, and task allocation. Therefore, the balanced values of accuracy, precision, recall, and F1-score are particularly important, as they indicate that neither false-positive nor false-negative detections dominate the perception process. This reduces unnecessary robot motions while minimizing the probability of missing recyclable objects, thereby increasing the overall reliability of the collaborative workstation.

The qualitative experiments further demonstrate the importance of post-processing within foundation-model-based perception. Although SAM generates highly accurate object proposals, multiple segmentation masks may correspond to different regions of the same physical object. The proposed nested-box filtering effectively removes these redundant detections, ensuring that each physical object is represented by a single grasp candidate. This significantly improves the stability of downstream localization and manipulation without modifying the underlying segmentation model.

Compared with conventional task-specific perception pipelines, the proposed SAM + MobileNetV2 architecture separates class-agnostic segmentation from lightweight classification, reducing the need for retraining the segmentation stage when new packaging categories are introduced. This modular design facilitates faster adaptation to changing waste streams while maintaining implementation simplicity, making it particularly suitable for small-batch industrial environments where packaging types and material composition frequently vary.

Beyond material classification, the proposed framework simultaneously estimates object geometry and damage level, providing semantic information that directly supports robotic decision-making. These additional perception outputs enable grasp-oriented reasoning and safety-aware task allocation by identifying objects that may require adapted manipulation due to sharp edges, fractures, or severe deformation, while geometric descriptors support the selection of appropriate grasp strategies.

### 5.2 Comparison with related work

Most existing robotic waste-sorting systems focus primarily on maximizing recognition accuracy or manipulation throughput in large-scale municipal material recovery facilities. Recent studies have demonstrated remarkable progress in deep-learning-based waste recognition using object detection, semantic segmentation, and instance segmentation networks. However, these systems are generally optimized for fully automated, high-throughput installations and rarely consider collaborative operation with human workers.

The proposed workstation addresses a different application domain. Rather than competing with large municipal sorting facilities, it targets small-batch industrial packaging waste streams, where flexibility, rapid reconfiguration, and safe human–robot collaboration are often more important than maximum throughput. Within this context, foundation-model-based perception provides a practical advantage because segmentation can be generalized to previously unseen object appearances without retraining dedicated segmentation networks.

Compared with conventional segmentation methods, the proposed SAM-based perception pipeline substantially reduces the effort required for dataset preparation while preserving accurate object localization. Furthermore, integrating segmentation, RGB-D localization, geometric reasoning, and damage assessment into a unified perception framework provides richer scene understanding than traditional classification-only approaches. This additional information is directly exploited by the task scheduler, allowing perception results to influence manipulation planning instead of serving solely as object labels.

The proposed scheduling architecture is also consistent with recent modular robotic planning frameworks that separate high-level task allocation from low-level trajectory generation. Similar to approaches such as RaapWaste, the proposed system decouples scheduling from robot motion planning, improving modularity and hardware independence. However, the present work extends this concept by incorporating human availability, object safety level, and damage assessment into the scheduling process, thereby explicitly supporting collaborative human–robot decision-making.

### 5.3 Human–robot collaboration

A distinguishing feature of the proposed workstation is that perception, scheduling, and manipulation are tightly integrated within a collaborative decision-making framework rather than operating as independent subsystems. Instead of maximizing robotic autonomy at all costs, the proposed architecture allocates tasks according to the capabilities of both the robot and the human operator.

This strategy is particularly beneficial when perception uncertainty increases or when waste objects exhibit characteristics that reduce manipulation reliability. Objects that are occluded, visually ambiguous, or outside the trained data distribution can be safely delegated to the human operator instead of forcing the robot to execute uncertain grasps. Conversely, damaged or potentially hazardous objects can be preferentially assigned to robotic manipulation, reducing worker exposure to sharp edges and fractured packaging.

Such capability reflects the emerging Industry 5.0 paradigm, where collaborative robots complement rather than replace human workers. Within the proposed workstation, robotic precision and repeatability are combined with human adaptability, enabling the system to maintain both operational flexibility and workplace safety under varying industrial conditions.

Although the collaborative scheduling framework was validated at the architectural level, the present study did not quantitatively evaluate metrics such as operator intervention frequency, task allocation efficiency, human workload, or collaborative productivity. These aspects represent an important direction for future experimental validation.

### 5.4 Industrial implications

The proposed workstation demonstrates that collaborative robotic sorting can be successfully adapted to industrial environments characterized by relatively small waste volumes, heterogeneous packaging types, and frequently changing production conditions. Such environments differ substantially from municipal material recovery facilities, where dedicated high-throughput equipment is economically justified.

One practical advantage of the proposed architecture is its modularity. The perception modules, scheduling algorithm, robot controller, and manipulation subsystem communicate through a ROS-based software framework, allowing individual components to be replaced without redesigning the entire system. Consequently, different collaborative robots, grippers, RGB-D cameras, or perception algorithms may be integrated according to application-specific requirements.

The incorporation of damage estimation and geometric reasoning further increases the practical applicability of the workstation. Beyond determining the appropriate recycling stream, these outputs influence grasp planning, manipulation safety, and collaborative task allocation, thereby transforming perception into an active decision-support component rather than a passive classification module.

Furthermore, the structured information generated during sorting—including object category, geometric descriptors, damage score, timestamps, and destination bins—creates opportunities for integration with digital manufacturing systems, production monitoring platforms, and traceability solutions. Such capability is particularly relevant for Industry 4.0 and circular-economy applications, where continuous monitoring of material flows is becoming increasingly important.

### 5.5 Limitations

Several limitations of the present study should be acknowledged. First, experimental validation was performed under controlled laboratory conditions using three representative packaging waste categories. Although these categories reflect common industrial packaging materials, they do not encompass the full diversity of real industrial or municipal waste streams.

Second, the laboratory experiments assumed mechanically preconditioned waste with limited overlap between consecutive objects. While this assumption reflects the intended small-batch industrial application, additional validation under progressively increasing clutter, partial occlusions, and irregular object arrangements is necessary to quantify perception robustness in more challenging operating conditions.

Third, the current evaluation focused primarily on validating the integrated perception–localization–manipulation pipeline rather than optimizing individual subsystem performance. Consequently, metrics such as recovery after failed grasps, scheduler efficiency, and long-term collaborative productivity were not quantitatively analyzed. The PointNet-based geometric reasoning module was evaluated qualitatively only, and its quantitative evaluation on a dedicated benchmark dataset remains part of future work.

Finally, although the proposed architecture is hardware-independent at the software level, experimental validation was performed using a single robotic platform, gripper configuration, and RGB-D sensing system. Future work will focus on adaptive grasp-point selection, closed-loop visual and force feedback, and trajectory adaptation to improve robustness against severe object deformation, object loss during transfer, and gripper-to-object misalignment.

Overall, the presented results demonstrate that integrating foundation-model-based perception, semantic scene understanding, and collaborative task scheduling into a unified robotic workstation constitutes a promising approach for flexible packaging waste sorting. Rather than maximizing automation alone, the proposed architecture emphasizes safe and adaptive cooperation between human operators and collaborative robots, thereby addressing an important gap between manual sorting and large-scale fully automated recycling systems.

## 6 Industrial relevance and potential economic implications

The proposed collaborative workstation addresses an important gap between manual waste sorting and highly automated material recovery facilities. While existing commercial solutions achieve excellent performance in high-throughput municipal recycling plants, their deployment typically requires substantial capital investment, dedicated infrastructure, and continuously large waste volumes. Such requirements often limit their applicability in industrial environments where packaging waste is generated intermittently, in relatively small quantities, and with frequently changing material composition.

Rather than maximizing throughput, the proposed system prioritizes flexibility, rapid reconfiguration, and safe human–robot collaboration. This design philosophy makes the workstation particularly suitable for manufacturing environments where packaging waste originates from production, logistics, assembly, or warehouse operations. In these scenarios, the ability to adapt the perception pipeline to new packaging types, integrate different robotic platforms, and modify task-allocation strategies may reduce application-specific engineering effort compared with fixed-purpose sorting systems.

A key industrial contribution of the proposed architecture is the integration of perception, semantic scene understanding, and collaborative task scheduling within a unified ROS-based framework. Beyond conventional object recognition, the perception subsystem provides damage estimation and geometric descriptors that directly support robotic manipulation and safety-aware decision making. This richer representation enables more informed routing strategies, adaptive grasp planning, and collaborative allocation of hazardous objects between human operators and robotic agents.

The generated perception data—including object category, geometric information, damage level, timestamps, and sorting destination—also create opportunities for integration with digital manufacturing platforms and traceability systems. Such information can support production analytics, quality monitoring, inventory reporting, and circular-economy indicators by providing continuous visibility into packaging waste streams throughout industrial operations.

From an Industry 5.0 perspective, the proposed workstation promotes human-centric automation rather than workforce replacement. The collaborative scheduling strategy combines robotic precision, repeatability, and safe handling of hazardous objects with the adaptability and decision-making capabilities of human operators. Consequently, automation is employed to complement human work, reduce exposure to potentially unsafe waste items, and improve overall workplace ergonomics.

Although a quantitative cost–benefit analysis and setup-time was outside the scope of the present study, several factors indicate potential industrial benefits. The workstation was implemented using an existing laboratory setup; therefore, representative installation and commissioning times could not be measured. The ROS-based and hardware-independent architecture allows integration of different commercially available cobots, cameras, and grippers, including lower-cost ROS-compatible platforms. However, these characteristics indicate only potential cost reductions and do not constitute a quantitative economic validation. A dedicated techno-economic assessment under industrial deployment conditions is therefore planned as future work.

## 7 Conclusion and future work

This paper presented a modular collaborative robotic workstation for small-batch packaging waste sorting that integrates perception, intelligent task allocation, and robotic manipulation into a unified framework. The proposed system combines foundation-model-based segmentation, lightweight object classification, RGB-D localization, damage assessment, and collaborative task scheduling to support flexible and safety-aware sorting under Industry 5.0 principles.

Experimental validation demonstrated the feasibility of the proposed architecture under controlled laboratory conditions. The perception pipeline achieved reliable recognition of representative packaging waste categories, while the integrated manipulation framework successfully executed continuous conveyor-based pick-and-place operations without interrupting material flow. Beyond conventional waste classification, the incorporation of geometric reasoning and damage estimation provides additional information for adaptive grasp planning and collaborative task allocation, enabling safer and more flexible human–robot interaction.

The principal contribution of this work is not the development of individual perception or manipulation algorithms, but their integration into a reconfigurable collaborative workstation specifically designed for small-batch industrial packaging waste sorting. The modular ROS-based architecture enables straightforward adaptation to different collaborative robots, end-effectors, perception modules, and industrial operating conditions, providing a flexible alternative to conventional fixed-purpose waste-sorting systems.

Several limitations remain. The current validation was conducted using a limited number of packaging waste categories under controlled laboratory conditions and did not include quantitative evaluation of collaborative scheduling efficiency or long-term industrial operation. Furthermore, highly cluttered waste streams, severe object deformation, contamination, and complex multi-object interactions remain challenging scenarios that require additional investigation.

Future work will focus on validating the workstation under representative industrial conditions using more diverse packaging waste streams and progressively increasing scene complexity. Particular emphasis will be placed on closed-loop visual and force feedback, adaptive grasp planning, quantitative evaluation of collaborative task allocation, and multi-robot coordination. In addition, future studies will investigate the techno-economic performance of the proposed workstation, including productivity, operator workload, and return on investment, to further assess its industrial deployment potential.

## Data Availability

The original contributions presented in the study are included in the article/supplementary material, further inquiries can be directed to the corresponding author.
